# Flat Anterior Chamber after Trabeculectomy in Secondary Angle-Closure Glaucoma with *BEST1* Gene Mutation: Case Series

**DOI:** 10.1371/journal.pone.0169395

**Published:** 2017-01-05

**Authors:** Yimin Zhong, Xinxing Guo, Hui Xiao, Jingyi Luo, Chengguo Zuo, Xiaobo Huang, Jingjing Huang, Lan Mi, Qingjiong Zhang, Xing Liu

**Affiliations:** State Key Laboratory of Ophthalmology, Zhongshan Ophthalmic Center, Sun Yat-sen University, Guangzhou, Guangdong, China; Bascom Palmer Eye Institute, UNITED STATES

## Abstract

**Purpose:**

Trabeculectomy has been regarded as a mainstay of initial treatment in eyes of angle closure glaucoma (ACG) with peripheral anterior synechia > 180° in the Chinese population while its efficacy in secondary ACG with *BEST1* gene mutation remains unclear. We set out to investigate the treatment outcome of trabeculectomy for secondary ACG in a group of patients with autosomal recessive bestrophinopathy (ARB).

**Methods:**

In this retrospective case series study, 8 secondary ACG patients with ARB and their 4 recruited family members underwent a thorough ophthalmic examination including best-corrected visual acuity, Goldmann applanation tonometry, gonioscopy, and fundus examinations. Ultrasound biomicroscopy, optical coherence tomography (OCT), ultrasound A-scan, B-scan, electro-oculography (EOG), Humphrey perimetry, fundus photography, fundus fluorescein angiography (FFA) and indocyanine green angiography (ICGA) were also performed. Blood samples were obtained in the patients and their available family members to analyze the variants of the *BEST1* gene. Trabeculectomy was performed in the 8 patients (15 eyes).

**Results:**

The age of onset varied from 13 to 38 years. The average axial length (AL) of the affected eyes was 21.82 ± 0.92 mm and the average anterior chamber depth (ACD) was 2.19 ± 0.29 mm. There was marked axial shallowing of the anterior chamber in all 15 eyes after trabeculectomy, and was not improved with potent mydriatics. The IOP was elevated in 3 eyes. Variable degree of yellowish subretinal deposits was observed in the posterior retina. The FFA showed punctuate or patched hyperfluorescence suggesting retinal pigment epithelium impairment. The ICGA demonstrated dilatation of choroidal vessels. The OCT revealed diffused neuroretinal detachment in the posterior and midperipheral retina, with intraretinal fluid collections, and hyperreflective subretinal accumulations. The average subfoveal choroidal thickness of the patients was 382.36 ± 80.09 μm. All the patients and enrolled family members carried mutation in *BEST1* gene.

**Conclusions:**

ARB is a rare condition with fundus manifestations mimicking various diseases. Careful discrimination should be taken to exclude any secondary causes for ACG before treatment. Concerning the high incidence of postoperative shallow anterior chamber, selection of filtering surgery should be very careful in these patients.

## Introduction

Angle-closure glaucoma (ACG) has a higher prevalence in the Chinese than the Caucasian population but rarely occurs in young individuals.[1] Often times ACG with earlier onset is associated with the development of ocular abnormalities.[2] Although usually treated with iridotomy in the Caucasian populations, for patients with peripheral anterior synechia (PAS)>180 degrees and without significant lens opacities, trabeculectomy is considered a mainstay of initial treatment in the Chinese population for its sustained intraocular pressure (IOP) lowering effect and long-term efficacy.[3]

The simultaneous occurrence of ACG or occludable anterior chamber angles, along with reduced axial length (AL), crowded anterior segments, severe hyperopia, and fundus abnormalities is extremely rare.[4, 5] Autosomal recessive bestrophinopathy (ARB) was first described by Burgess et al. in 2008, with mutations in *BEST1* gene and an association of hypermetropia and angle closure.[6] Due to its mimical phenotypes to other diseases such as multifocal *Best* disease, adult-onset vitelliform macular dystrophy (VMD), exudative polymorphous vitelliform maculopathy, etc., molecular genetic confirmation of mutations in *BEST1* is necessary in confirming the diagnosis.[7, 8]

There has been limited knowledge on ACG in ARB subjects, with scarce reports on disease presentation and treatment options.[9–11] We hereby report a group of ARB patients with ACG and their treatment outcomes. All of these subjects underwent trabeculectomy and developed refractory flat anterior chamber postoperatively. In this study, we presented the clinical characteristics, and investigated the potential relationship of the postoperative shallow anterior chamber and these characteristics.

## Materials and Methods

### Study Population

In this retrospective, observational case-series study, 8 consecutive patients with ARB and ACG diagnosed at the Zhongshan Ophthalmic Center (ZOC, Guangzhou, China) from Nov 2005 to Sep 2014 were included. Four family members of these patients with similar fundus changes were also recruited. This study adhered to the tenets of the Declaration of Helsinki. Written informed consent was obtained from all subjects or their legal guardians on behalf of the minors enrolled in this study. Ethical approval was given by the institutional review board of ZOC.

## Methods

All subjects (8 patients and 4 family members) underwent a series of ophthalmic examinations in both eyes. Clinical information was reviewed to detail the demographic characteristics, clinical manifestations and surgical outcomes. These included best-corrected visual acuity (BCVA), non-cycloplegic refraction, slit-lamp biomicroscopy, intraocular pressure (IOP) measurement using Goldmann applanation tonometry, and detailed funduscopy. Examination of filtering status excluded bleb leakage and overfiltraion by slit-lamp biomicroscopy. Gonioscopy was performed with a Goldmann one-mirror lens (Haag-Streit, Bern, Switzerland) with the anterior chamber angle graded by Scheie classification system. Anterior chamber depth (ACD) was measured by ultrasound biomicroscopy (UBM, Sower, Tianjin, China). AL was measured by ultrasound A-scan (Cinescan A/B scan, Quantel Medical, Clemon, France). B-scan was performed to exclude any choroidal effusion or hemorrhage. Visual field analysis was conducted by automated perimetry (Humphrey 750; Carl Zeiss Meditec, Inc., Dublin, CA) using 30–2 SITA-standard strategy. Fundus color photography was performed with a fundus camera (Kowa, Tokyo, Japan). Fundus fluorescein angiography (FFA) and indocyanine green angiography (ICGA) (HRA 2; Heidelberg Engineering, Heidelberg, Germany) were performed.

Optical coherence tomography (OCT) imaging was conducted in all patients and the family members by Spectralis OCT (Heidelberg Engineering, Heidelberg, Germany) using line scan protocol (30°) or Stratus OCT (Carl Zeiss, Dublin, CA) using 5-line scan protocol (6mm scan length with 0° or 90° scanning angle). Subfoveal choroid was scanned with enhanced depth imaging (Heidelberg Engineering, Heidelberg, Germany) and the thickness was measured. Subfoveal choroid thickness (SFCT) was defined as the perpendicular distance from the outer portion of the hyper-reflective line corresponding to the retinal pigment epithelium (RPE) to the sclerochoroidal interface. The manual caliper function in the Heidelberg Spectralis OCT software was applied. The average values from the vertical and horizontal section OCT image were obtained for analysis.

Electro-oculography (EOG) (RetiPort32, Roland Consult, Wiesbaden, Germany) was performed in all patients according to the guidelines of the International Society for Clinical Electrophysiology of Vision (ISCEV).[12] Arden ratios below 1.8 were rated as pathologic.

### Genetic Analysis

Variants of the *BEST1* gene were analyzed in the patients and their available family members. Genomic DNA samples were extracted from peripheral blood leukocytes according to established protocols from all the individuals mentioned above. The ten exons of the *BEST1* gene and flanking intronic regions were amplified by PCR and sequenced by ABI 3730XL DNA sequencer. Variants are considered as functionally highly deleterious if they meet the following criteria: (1) Minor allele frequency <0.01 in the 1000 Genomes and ExAC; 2) Predicted to be damaged in SIFT (available in the public domain at http://sift.jcvi.org/) or PolyPhen-2 tools (available in the public domain at http://genetics.bwh.harvard.edu/pph2/); 3) Mutations reported in previous studies are also considered pathogenic if not detected in normal individuals; 4) Corresponding with the law of coseparation in family members.

### Surgical Procedure

Trabeculectomy was performed in 8 patients (15 eyes) by glaucoma specialists in ZOC. A limbus based conjunctival flap was prepared, and a square 4×3mm scleral flap (half the scleral thickness) was dissected. Mitomycin C of 0.25 mg/ml was applied for 3 minutes and then washed off with balanced saline solution. After a small trabecular tissue was incised and peripheral iridectomy was made, the scleral flap was tightly closed with 4 sutures (2 releasable and 2 permanent) to ensure formation of the anterior chamber.[13] No complications were observed during the operation.

## Results

### Demographic Characteristics and Clinical Examination

All patients were bilaterally involved. The average age at onset was 26.6 ± 8.7 (range, 13~38) years, and there were 3 female and 5 male patients. The BCVA ranged from 20/30 to finger counting. The spherical refraction of the affected eyes ranged from +0.50 to +3.00 diopters. The average IOP was 31.3 ± 3.7 (range, 25.0~36.0) mmHg with multiple antiglaucoma medications before operation. Narrow anterior chamber angle (Grade Ⅲ~Ⅳ in Scheie classification) was observed in all patients on static gonioscopy; with dynamic gonioscopy, the median degree of PAS was 300 (range, 210~360). The glaucomatous visual field damage was at moderate-stage (MD between −6 dB and −15 dB) in 5 eyes (31.3%) and advanced-stage (MD≤−15 dB) in 11 eyes (68.8%).

Ocular biometry showed mean ACD of 2.19 ± 0.29 (range, 1.76~2.65) mm, which was shallower than the normal range of 2.68~3.54 mm;[14] mean AL of 21.82 ± 0.92 (range, 20.10~23.29) mm, which was shorter than the normal range of 22.00~24.50 mm. [14] SFCT measurements revealed a thickened choroidal thickness of 382.36 ± 80.09 (range, 222~502) μm in the affected eyes, as compared to normal range of 294.63 ± 75.90 μm.[15, 16]

### Surgical Outcome and Postoperative Treatment

**Fig 1:**
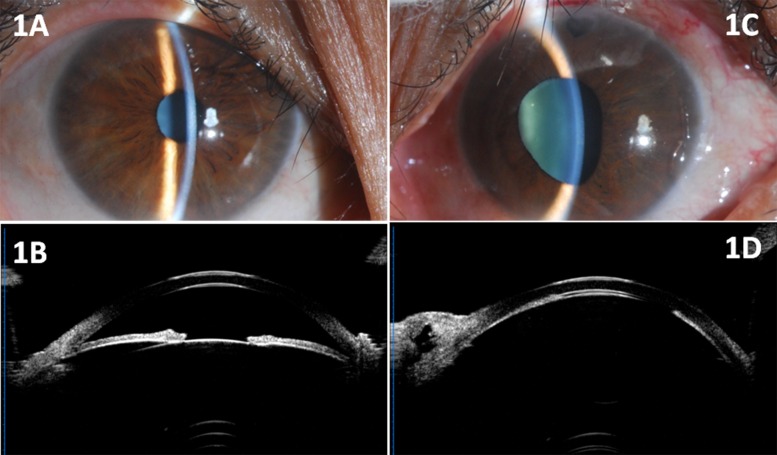
Anterior segment photograph and UBM images of a 46-year-old female patient. (1A, 1B) Photograph and UBM showed relatively shallow anterior chamber of the right eye. (1C) Color photograph showed flat anterior chamber and moderately dilated pupil of the left eye. (1D) UBM findings showed flat anterior chamber (nearly corneo-lenticular touch) in the left eye one month after trabeculectomy.

Trabeculectomy was performed in 8 patients (15 eyes). Surgery was spared in 1 eye because of controlled IOP with medications. Axial shallowing of the anterior chamber developed in all 15 eyes as early as the first week or up to 8 months after trabeculectomy (Fig 1). The axial ACD was decreased to 0.62 ± 0.27 (range, 0~0.91) mm. The average IOP on the first day after trabeculectomy was 15.3 ± 7.2 (range, 8.0~35.0) mmHg. Among these eyes, 3 (20%) eyes showed elevated IOP of 22~35 mmHg and 12 eyes (80%) retained IOP within the normal range (12.6 ± 3.8 mmHg). During the follow-up, the elevated IOP was controlled by tropical hypotensive drugs, while flat anterior chamber persisted although potent cycloplegics, topical and systemic corticosteroids were administered.

### Ocular Imaging

In order to investigate the nature of the fundus changes and its potential relationship with post-operative shallow anterior chamber, further examinations were performed after the operation in these patients, including dilated fundus photography, FFA, ICGA, OCT and EOG.

**Fig 2:**
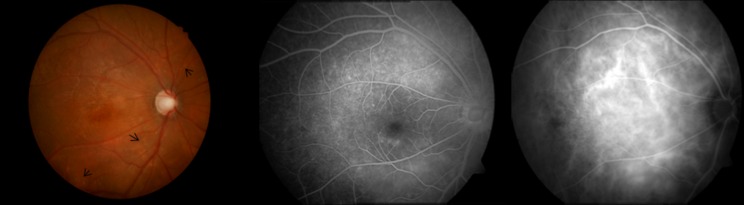
Images of a 22-year-old male patient. (2A) Fundus photograph demonstrating increased cup-to-disc ratio, RPE irregularities in the macula and the surrounding retina, with yellow-white subretinal deposits (black arrows). No obvious macular edema was observed. (2B) FFA showing punctuate or patched hyperfluorescence in the macula and the posterior retina. (2C) ICGA showing markedly dilated choroidal vessels in the posterior pole.

Fundus photograph demonstrated increased cup-to-disc ratio in all examined eyes, and scattered yellowish subretinal depositions in the posterior retina in 14 out of 16 eyes (87.5%) (Fig 2A). FFA showed punctuate or patched hyperfluorescence in the macula and/or the posterior retina from the early stage of angiography, mostly without leakage, and largely faded at the late stage, suggesting impairment of the RPE (Fig 2B). ICGA of the affected eyes demonstrated marked dilatation and increased permeability of the choroidal vessels in the macula and the surrounding posterior retina in both eyes (Fig 2C). Abnormal EOG (decreased Arden ratio) was found in all the patients.

**Fig 3:**
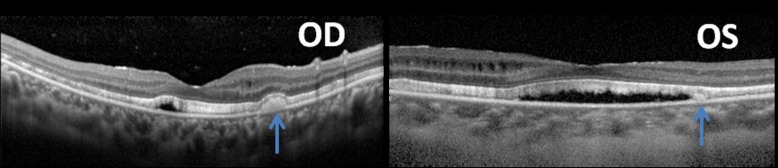
OCT images of a 23-year-old female patiento. OCT images of right and left eyes showing thinning of the retinal nerve fiber layer, diffused serous neuroretinal detachment, parafoveal intraretinal fluid collections, hyperreflective subretinal accumulations (blue arrows), elongated and thickened photoreceptor outer segment layer.

The OCT findings of the affected eyes were summarized as the following (Fig 3): (1) Diffused shallow detachment of the neuroretinal layer was found in both eyes in all the patients; (2) Cystoid fluid collections in the macula. These could be further described as two features: 1) cystoid macular edema (CME) (4 patients, 6 eyes); and 2) cystoid fluid collections outside the fovea (8 patients, 16 eyes); (3) Scattered hyperreflective subretinal accumulations in the macula or in the posterior retina (8 patients, 16 eyes); (4) Thickening of the photoreceptor outer segment layer (5 patients, 10 eyes).

### Clinical Features of the Family Members

Ophthalmic examinations were performed in the available family members of the patients. Among these, 4 subjects had similar fundus changes. There were 3 males and 1 female. The age at onset was 6, 26, 33 and 49 years, respectively. Short AL and shallow anterior chamber were found in both eyes. Three males had ACG, and the female presented with angle-closure suspect. OCT demonstrated diffused subretinal fluid and intraretinal fluid in bilateral eyes.

### Genetic Characteristics

All patients were found to carry mutations in the *BEST1* gene (Table 1). There were 4 novel mutations. Six patients were found to carry compound heterozygous mutations, and 2 patients (Patient 2 and Patient 7) carried homozygous mutations. The second mutation c.247+2T>G of patient 4 was predicted to affect mRNA splicing of the *BEST1* gene in BDGP (available in public domain at http://www.fruitfly.org/seq_tools/splice.html) and HSF tools (available in the public domain at http://www.umd.be/HSF3/HSF.html).

**Table 1:** *BEST1* mutations identified in the patients.

Patient	Age at onset	Mutation	Protein Effect	SIFT	Polyphen-2	Reported or not
1	13	c.38G>A/c.763C>T	p.R13H/p.R255W	T/D	B/PrD	Reported/reported
2	29	c.763C>T/c.763C>T	p.R255W/p.R255W	D/D	PrD/PrD	Reported
3	21	c.389G>T/c.488T>G	p.R130L/p.M163R	D/D	PrD/PrD	Reported/reported
4	19	c.842T>C/c.247+2T>G	p.F281S/Splicing defect	D/ NA	PrD/ NA	Novel/novel
5	38	c.1070C>T/c.1550C>G	p.A357V/p.S517*	D/NA	B/NA	Novel/novel
6	25	c.764G>A/c.830C>T	p.R255Q/p.T277M	T/D	PrD/PrD	Novel/reported
7	35	c.1066C>T/ c.1066C>T	p.R356* /p.R356*	NA/ NA	NA/ NA	Reported
8	33	c.764G>A/c.763C>T	p.R255Q/p.R255W	D/D	PrD/PrD	Novel/reported

T, Tolerated; D, Damaging; PrD, Probably damaging; B, Benign; NA, No applicable for variants other than amino acid substitution.

## Discussion

The patients in this case series demonstrated a variable degree of yellowish subretinal depositions in the posterior retina. On OCT, diffused flat detachment of the neuroretinal layer was observed in the posterior and midperipheral retina, with intraretinal fluid and hyperreflective subretinal spots or protrusions. The FFA showed punctuate or patched hyperfluorescence in the macula and/or the posterior retina from the early stage of angiography, and largely faded at the late stage, suggesting RPE loss. The EOG showed decreased Arden ratio. These clinical findings, combined with the genetic characteristics, are compatible with the diagnosis of autosomal recessive bestrophinopathy (ARB).[6–8, 14, 17–20] Although in 4 patients, the OCT images were obtained after the operation, we consider that these pathological changes, e.g. serous retinal detachment, or cystoid macular edema were not caused by the operation itself, for there was no ocular hypotension when the OCT was performed. Moreover, the similar fundal changes were found in some family members of these patients. And these findings were also detected in other 4 patients before trabeculectomy. The genetic analysis revealed *BEST1* gene mutation in all the patients.

The *BEST1* gene encodes the bestrophin-1 protein that localizes to the RPE.[21, 22] Bestrophinopathies caused by *BEST1* gene mutations are found to be associated with reduced AL, hyperopia and a high incidence of ACG.[6, 8, 9, 23, 24] Up to 50% of ARB patients are at risk of angle closure glaucoma developing as a result of narrow anterior chamber angles.[6, 14] But the role of bestrophin-1 in the development of ACG has not been elucidated. Treatment of ACG secondary to ARB might be rather difficult. Yttrium–aluminum–garnet (YAG) laser peripheral iridotomy alone may not be sufficient in lowering the IOP.[14] And cataract extraction in the case of persistent high IOP was not effective to control IOP as it could not open the anterior chamber angle to a sufficient degree.[14] These patients may have a dysgenesis of the anterior segment that also affects the trabecular meshwork, in addition to a shallow anterior chamber and occludable anterior chamber angles.[14]

All these patients presented for the first time to the ophthalmologists as ACG. As the fundal changes of the patients were difficult to identify with small pupils, some were misdiagnosed as primary angle-closure glaucoma (PACG). In China, for PACG patients with PAS >180 degrees and without significant lens opacities, trabeculectomy is usually a mainstay of initial treatment.[3] So before the nature of the fundal changes was fully recognized, trabeculectomy was selected in these moderate- to advanced-staged glaucoma patients. Although there were no complications during the operations, all patients developed refractory shallow anterior chamber after trabeculectomy. Unlike typical malignant glaucoma, only 20% patients showed increased IOP, while most patients demonstrated normal IOP.

Shallower ACD and shorter AL may be related to the development of flat anterior chamber after trabeculectomy in ACG.[25] Although this group of patients showed a short AL, the average ACD of these patients (2.19 ± 0.29 mm) was much deeper than that of malignant glaucomatous patients (1.86±0.39 mm).[26] Therefore we suspect that the fundal changes of these patients may play a role in the development of postoperative refractory flat anterior chamber. The pre-existed extensive subretinal and intraretinal fluid accumulations, caused by impairment of normal fluid homeostasis by the RPE due to bestrophin-1 dysfunction, [6, 23, 27, 28] may raise pressure difference between the posterior chamber/vitreous cavity and the anterior chamber, especially when filtering exists. This might easily cause forward lens movement and refractory shallow anterior chamber after trabeculectomy.

Choroidal expansion could be another possible contributor in the pathogenesis of flat anterior chamber after trabeculectomy.[25, 29] The underlying cause of the diffused dilatation of choroidal capillaries in the posterior pole revealed by ICGA in these patients is unknown. However, vasodilatation of the choroid might possibly lead to choroid expansion. Choroidal expansion is further documented by increased choroidal thickness in these patients.

In conclusion, as the etiology of angle closure in young people is quite different from that in the older population and is typically associated with structural or developmental ocular anomalies, we should perform careful examinations, especially fundus examinations to exclude any secondary causes.[2] This group of ACG patients with fundus changes should be differentiated from PACG and we recommend filtering surgery avoided in these patients.

As we known, this is the first report about the treatment outcome of trabeculectomy for secondary ACG in ARB patients. As the current study is a retrospective case series and some of the ophthalmic examinations, e.g. FFA, ICGA, OCT and SFCT, were performed after trabeculectomy, biases would appear in the analysis of the results. The number of this case series is small. Further studies are needed to determine the characteristics of secondary glaucoma associated with ARB and investigate the effects of different therapeutic regimens.

## Abbreviations

ACG: angle-closure glaucoma
PAS: peripheral anterior synechia
IOP: intraocular pressure
AL: axial length
ARB: autosomal recessive bestrophinopathy
VMD: vitelliform macular dystrophy
ZOC: Zhongshan Ophthalmic Center
BCVA: best-corrected visual acuity
ACD: anterior chamber depth
UBM: ultrasound biomicroscopy
FFA: fundus fluorescein angiography
ICGA: indocyanine green angiography
OCT: optical coherence tomography
SFCT: subfoveal choroid thickness
RPE: retinal pigment epithelium
EOG: electro-oculography
CME: cystoid macular edema
YAG: yttrium–aluminum–garnet
PACG: primary angle-closure glaucoma
